# Detection of Non-PCR Amplified *S. enteritidis* Genomic DNA from Food Matrices Using a Gold-Nanoparticle DNA Biosensor: A Proof-of-Concept Study

**DOI:** 10.3390/s120810487

**Published:** 2012-08-02

**Authors:** Sylvia A. Vetrone, Michael C. Huarng, Evangelyn C. Alocilja

**Affiliations:** 1 Department of Biology, Whittier College, 13406 E. Philadelphia St., Whittier, CA 90608, USA; E-Mail: svetrone@whittier.edu; 2 Department of Biosystems and Agricultural Engineering, 213 Farrall Hall, Michigan State University, East Lansing, MI 48824, USA

**Keywords:** biosensor, gold-nanoparticles, DNA probe, genomic DNA, food matrix

## Abstract

Bacterial pathogens pose an increasing food safety and bioterrorism concern. Current DNA detection methods utilizing sensitive nanotechnology and biosensors have shown excellent detection, but require expensive and time-consuming polymerase chain reaction (PCR) to amplify DNA targets; thus, a faster, more economical method is still essential. In this *proof-of-concept* study, we investigated the ability of a gold nanoparticle-DNA (AuNP-DNA) biosensor to detect non-PCR amplified genomic *Salmonella enterica* serovar Enteritidis (*S. enteritidis*) DNA, from pure or mixed bacterial culture and spiked liquid matrices. Non-PCR amplified DNA was hybridized into sandwich-like structures (magnetic nanoparticles/DNA/AuNPs) and analyzed through detection of gold voltammetric peaks using differential pulse voltammetry. Our preliminary data indicate that non-PCR amplified genomic DNA can be detected at a concentration as low as 100 ng/mL from bacterial cultures and spiked liquid matrices, similar to reported PCR amplified detection levels. These findings also suggest that AuNP-DNA biosensors are a first step towards a viable detection method of bacterial pathogens, in particular, for resource-limited settings, such as field-based or economically limited conditions. Future efforts will focus on further optimization of the DNA extraction method and AuNP-biosensors, to increase sensitivity at lower DNA target concentrations from food matrices comparable to PCR amplified DNA detection strategies.

## 1. Introduction

**Figure 1.**
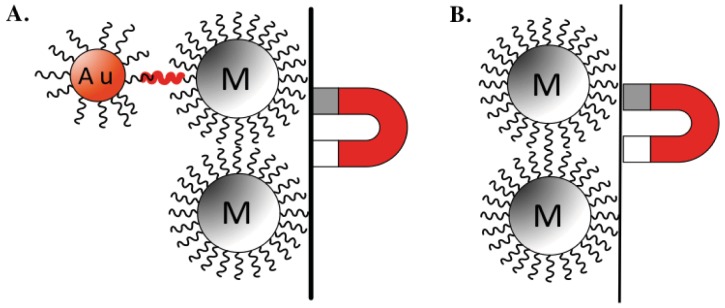
Schematic of AuNP-DNAt-MNP sandwich structure. (**A**) The presence of specific DNAt (red wavy bar) will covalently bind with DNAt specific probes (black wavy lines) on AuNP (red Au labeled bead) and the MNP (grey M labeled bead), allowing the collection of AuNP. (**B**) Absence of specific DNAt will lead to the failure of AuNP collection (image used with permission from Michael J. Anderson at Michigan State University, 2012).

The 2001 distribution of *Bacillus anthracis* (*B. anthracis*) through the United States postal system, which resulted in 22 cases of anthrax exposure and five deaths, brought much needed awareness to the significant effect of bacterial pathogens on public health [1]. Bioterrorism became a reality and identified critical needs in prevention, protection, and mitigation for homeland security, especially within the areas of food, water, and agricultural safety. To date, there are multiple commercially available methods for the detection of pathogenic agents, although none adequately comply with governmental food safety standards [2]. In addition, many of these methods utilize expensive reagents and equipment, and require a lengthy turn around period, as they often necessitate long incubation or detection times over a period of days [3,4]. Toward this end, much attention has been directed to the development of rapid, sensitive, low cost, portable biosensors for the biological detection of pathogens, such as those incorporating the use of nanoparticles for DNA detection [5–16]. Specifically, these analytical devices integrate a biological sensing element with a transducer to quantify a biological event, such as the presence of pathogenic microorganisms within a liquid or solid matrix, into an electrical output. The detection relies on the immobilization of single stranded DNA (ssDNA) probes that are complementary and specific for a DNA sequence of the pathogenic target, on two separate surfaces: magnetic nanoparticles (MNPs) and gold nanoparticles (AuNPs) (Figure 1). MNPs are used to extract the DNA target from the sample while AuNPs are used to report the sandwich hybridization. AuNPs are used here because of their ease of production and functionalization [17,18]. The nanoparticles conjugated with ssDNA probes specific for the pathogenic target of interest are then hybridized with the DNA test sample, isolated using magnetic separation, and detected through electrochemical analysis [16,19–21]. While biosensors using this detection strategy have been shown to detect specific DNA fragments from various pathogens [22–24], many of these biosensors have only been tested for the detection of purified and polymerase chain reaction (PCR) amplified DNA targets (DNAt), and not from genomic DNAt extracted from pure bacterial samples or contaminated food matrices. Similar to some of the limitations of commercially available detection strategies, PCR is often criticized for its complex, expensive, time-consuming, and labor-intensive procedure requirements. Consequently, the need of PCR for biosensor detection of pathogenic DNAt is greatly restrictive for both field-based and resource limited settings, resulting in the increased need for a PCR-independent biosensor detection methods.

In this *proof-of-concept* study, we investigated the ability of a AuNP-DNA biosensor to detect non-PCR amplified genomic *Salmonella enterica* serovar Enteritidis (*S. enteritidis*) DNAt, as this *Salmonella* species is most frequently reported as the cause of foodborne illnesses in the United States [25]. Specifically, we designed ssDNA probes specific for the DNA insertion element (*Iel*) of *S. enteritidis*, and functionalized/conjugated them onto MNPs and AuNPs. The *S. enteritidis* DNA used as DNAt was directly extracted from pure culture, mixed culture, and contaminated liquid food matrix, and then directly hybridized into sandwich-like structures consisting of MNPs/genomic DNAt/AuNPs (Figure 1). Sandwich structures were analyzed for the presence of the non-PCR amplified genomic DNAt through the direct electrochemical detection of gold voltammetric peaks using differential pulse voltammetry (DPV).

## 2. Materials and Methods

### 2.1. Preparation of Gold Nanoparticles (AuNPs)

Gold nanoparticles were synthesized by a chemical reduction method as published by Hill and Mirkin [26]. Briefly, 1 mM hydrogen tetrachloroaurate (III) trihydrate was prepared with pure type I water. The gold solution was then covered and heated for 15 min, during which 38.8 mM sodium citrate was added, resulting in a color change from yellow to clear, to black, to purple and finally deep red. The solution was then allowed to cool and stored at room temperature until needed.

### 2.2. DNA and Oligonucleotide Target Probes for AuNPs, MNPs, and PCR Amplified Target DNA

All oligonucleotides were synthesized by Integrated DNA Technologies Inc. (Coralville, IA, USA) as previously published [15]. In this study, PCR amplified complementary DNA targets were used for comparison or as negative controls. The creation of the PCR amplified DNAt positive control and the ssDNA probes for *S. enteritidis* were established by designing primers against the *insertion element* (*Iel*) gene of *S. enteritidis*, to ensure specificity to our *S. enteritidis* DNAt and decrease non-specific hybridization to non-*S. enteritidis* DNAt [27]. The following nucleotide sequences were used to create the ssDNA probes specific for *S. enteritidis* to be conjugated onto the magnetic and gold nanoparticles: ssDNA probe on Au-NPs: 5′-AATATGCTGCCTACTGCCCTACGCTT-SH-3′ (position: 919∼944), ssDNA probe on MNPs: 5′-SH-TTTATGTAGTCCTGTATCTTCGCCGT-3′ (position: 661∼686). From isolated *S. enteritidis* genomic DNA, we created a PCR amplified DNAt positive control using the following two primers: forward primer: 5′-CTAACAGGCGCATACGATCTGACA-3′ and reverse primer: 5′-TACGCATAGCGATCTCCTTCGTTG-3′. The creation of the PCR amplified non-specific DNAt (NS-DNA) used as negative control was made from isolated *Bacillus anthracis (B. anthracis)* genomic DNA, and used at a concentration of 0.1 ng/μL (100 ng/mL). *B anthracis* primers were designed against the *pagA* gene (accession number, M22589) using the following two primers: forward primer: 5′-AAAATGGAAGAFTGAGGGTG-3′ and reverse primer: 5′-CCGCCTTTCTACCAGATTTA-3′.

### 2.3. Detection of Pathogenic Target DNA via Hybridization with Functionalized AuNPs and MNPs

Functionalization of AuNPs and MNPs were executed as previously published [15]. Hybridization of DNAt samples was also executed as previously published [15]. In summary, extracted non-PCR amplified genomic DNAt (diluted to concentrations of 0.1 ng/μL, 1 ng/μL, and 3 ng/μL), PCR amplified DNAt, PCR amplified NS-DNA (negative control, 0.1 ng/μL) or H_2_O (blank), were denatured at 95 °C for 10 min with a thermocycler (Mastercycle *personal*, Eppendorf, Hamburg, Germany). Each denatured sample was mixed with functionalized MNPs and assay buffer and incubated at 45 °C for 1 h on a rotor shaker (model HS-101, Amerex Instruments, Inc., Lafayette, CA, USA). Following the incubation, MNP-DNAt complexes were washed and resuspended with assay buffer, and functionalized AuNPs were added. The mixtures were incubated at 45 °C for 2 h with constant rotation. Next, the hybridized sandwiched samples (AuNPs-DNAt-MNPs) were washed and resuspended with assay buffer, transferred to screen-printed carbon electrodes (SPCEs, Gwent Electronic Materials, Ltd., Pontypool, UK), and dried for 30 min at room temperature. Once dry, 1 M HCl was added directly to all SPCEs to dissolve the AuNPs and generate Au^3+^ ions. DPV was performed using a desktop potentiostat (Potentiostat/galvanostat model 263A, Princeton Applied Research, Oak Ridge, TN, USA) from 1.25 V to 0.0 V (with a step potential of 10 mV, modulation amplitude of 50 mV, and scan rate of 33.5 mV/s) to generate voltammograms produced by the reduced gold ions on the SPCE. A SPCE containing only HCl was used for establishing the baseline background noise. For each sample tested (control or experimental), two samples (duplicates) were made and assayed to garner an average DVP readout per sample.

### 2.4. Bacterial Strain Culturing and Liquid Food Matrix Spiking

All bacterial cultures were obtained from the Michigan State University Food Safety and Toxicology Center. Individual pure bacterial cultures (PBC) of *Salmonella enterica* serovar Enteritidis (*S. enteritidis*, strain S-64), *Shigella boydii* (*S. boydii*), and *Escherichia coli* (*E. coli* O157:H7) were grown in Lysogeny broth (LB) overnight at 37 °C with gentle shaking. The next day, a serial dilution of each primary culture was prepared creating four concentrations estimated at 1.0^4^ cfu/mL, 1.0^5^ cfu/mL, 1.0^6^ cfu/mL, and 1.0^8^ cfu/mL (cfu, colony forming units). From these dilutions, 1.0 mL of these samples were: (1) taken for direct DNA extraction or (2) directly used to contaminate/spike 9 mL of liquid food matrix (basic = 2% milk or acidic = 100% orange juice). The contaminated liquid food matrix samples were allowed to incubate at room temperature for 15 min and then 1.0 mL of each contaminated sample was used for DNA extraction. To create a mixed bacterial culture (MBC), 1.0 mL of each PBC (*S. enteritidis, S. boydii*, and *E. coli*) at a concentration of 10^5^ cfu/mL, were mixed together and a volume of 1.0 mL of that mixture was used directly for DNA extraction or to contaminate/spike 9 mL of liquid food matrix.

### 2.5. DNA Extraction from Pure Culture, Mixed Bacterial Culture, and Bacterial-Spiked Food Matrix

Two DNA extraction methods were used in this study, TRIzol^®^ (# 15596-018, Life Technologies Corp., Grand Island, NY, USA) and phenol/ethanol [28]. Quantification and purity of isolated DNA samples were assessed using the NanoDrop 1000 Spectrophotometer (Thermo Scientific, Ashville, NC, USA).

#### TRIzol^®^ extraction

DNA samples extracted using TRIzol^®^ were performed according to the manufacturer's instructions. Briefly, 1.0 mL of bacterial culture (PBC or MBC) or liquid food matrix contaminated/spiked with bacterial culture was centrifuged at 13,000 g for 10 min at 4 °C. Supernatants were discarded, pellets resuspended in 1.0 mL of TRIzol^®^, quickly vortexed, and incubated at room temperature for 5 min. Next, 200 μL of chloroform was added, shaken vigorously by hand for 15 s, and incubated at room temperature for 3 min. Samples were centrifuged at 12,000 g for 15 min at 4 °C, the upper aqueous (RNA) supernatant was discarded. Next, 300 μL of 100% ethanol was added to the samples, mixed by inversion, and incubated for 3 min at room temperature. Samples were then centrifuged at 2,000 g for 5 min at 4 °C, the phenol-ethanol supernatant was discarded, and pellets were washed twice with 1.0 mL of 0.1 M sodium citrate in 10% ethanol by incubating for 30 min at room temperature followed by spinning down at 2,000 g for 5 min at 4 °C. All samples were then resuspended in 1.5 mL of 75% ethanol and incubated at room temperature for 20 min, mixing intermittently, followed by centrifugation at 2,000 g for 5 min at 4 °C. Pellets were then allowed to air-dry for 15 min, and resuspended in 300 μL of 8 mM sodium hydroxide (NaOH) followed by a final centrifugation at 12,000 g for 10 min at 4 °C. The supernatants were then transferred into new tubes and stored at 4 °C until needed.

#### Phenol/Ethanol Extraction

DNA samples isolated using the standard Phenol/ethanol method was performed as previously published [28]. Briefly, 1.0 mL of the MBC or liquid food matrix spiked with MBC was centrifuged at 13,000 g for 10 min at 4 °C. The supernatant was then discarded and the pellet was resuspended in 500 μL of TE Buffer (5.0 mL of 1 M Tris pH 8.0, 1.0 mL of 0.5 M EDTA pH 8.0 in 494.0 mL of type I water). Pellets were then broken down by the addition of 10 μL of lysozyme (#L6876, Sigma-Aldrich, St. Louis, MO, USA) and incubated for 15 min on ice. Next, 25 μL of Proteinase K (#P2308, Sigma-Aldrich) was added and samples were incubated for 10 min at 55 °C with shaking. A volume of 30 μL of 20% sodium dodecyl sulfate (SDS) was then added and samples were incubated for 1 h at 55 °C with shaking. Following the incubation, 500 μL of phenol (#P4557, Sigma-Aldrich) was added and samples were incubated for 5 min at 45 °C with shaking followed by a cool down for 5 min at room temperature. Samples were then centrifuged at 13,000 g for 10 min at 4 °C, and the upper aqueous (DNA) supernatant was transferred into a new tube. Samples then received the addition of 95% ethanol (2 volumes relative to the sample volume), mixed by inversion, incubated for 10 min on ice and centrifuged at 13,000 g for 10 min at 4 °C. The ethanol was carefully removed leaving behind approximately 10 μL to avoid removing the pellet. Samples were once again centrifuged at 13,000 g at room temperature, the supernatant was removed and allowed to air-dry for 15–20 min with the tube open at room temperature. The DNA pellets were then resuspended in 50 μL of sterile TE buffer, incubated at 37 °C for 10 min, and stored at 4 °C until needed.

## 3. Results

### 3.1. Extraction of Genomic DNA from Bacterial Culture

In an effort to move away from using PCR amplification of DNAt samples, we used two different genomic DNA extraction methods, the commercially available reagent TRIzol^®^ and the more economical phenol/ethanol extraction method [28], that can be used under resource limited settings. Using these two methods, genomic DNA to be used as DNAt was extracted from 1.0 mL aliquots of PBC, MBC, and liquid food matrix samples (2% milk or 100% orange juice) spiked with PBC or MBC. Both methods resulted in a useful yield of DNA, ranging from 232–461 ng/μL.

### 3.2. AuNP-DNA Biosensor Detection of Genomic DNA Extracted from PBC and MBC

**Figure 2.**
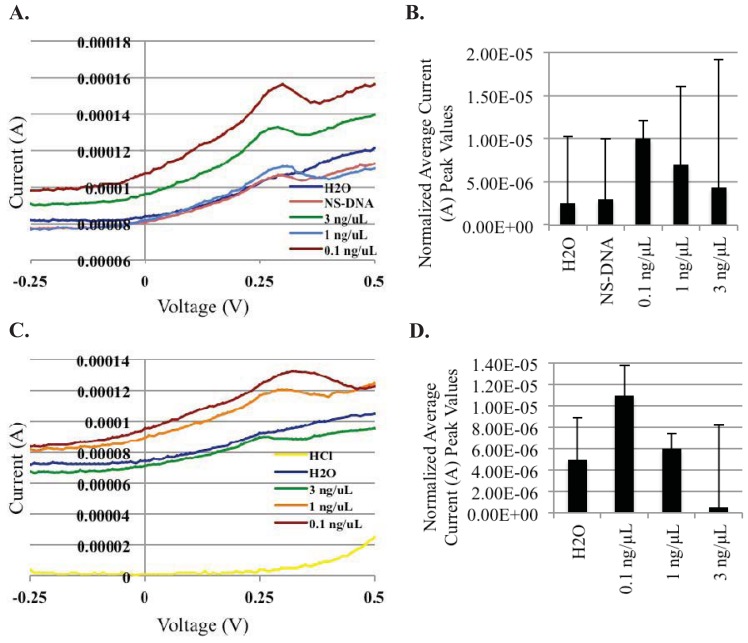
AuNP-DNA biosensor detection of non-PCR amplified *S. enteritidis* genomic DNAt. (**A**) Differential pulse voltammogram of PCR-amplified DNAt from *S. enteritidis* at various concentrations. (**B**) DNAt concentration *vs.* average differential current peak values from the voltammogram in A. (**C**) Differential pulse voltammogram of TRIzol^®^ extracted *S. enteritidis* genomic DNAt at various concentrations. (**D**) DNAt concentration *vs.* average differential current peak values from the voltammogram in C. H_2_O, blank control; NS-DNA, non-specific PCR amplified *B. anthracis* DNAt (0.1 ng/μL) negative control; HCl, 1 M hydrogen chloride. Graphs represent the average value of duplicate samples for each condition. Error bars represent the standard deviation of the mean.

With the successful extraction of genomic *S. enteritidis* DNAt using both extraction methods, we next explored the ability of the AuNP-DNA biosensor system to detect the non-PCR amplified genomic DNAt products. For these experiments, extracted genomic DNAt samples were diluted to three concentrations (3 ng/μL, 1 ng/μL, and 0.1 ng/μL), denatured, and then mixed with functionalized magnetic MNPs and AuNPs (both contained immobilized/conjugated ssDNA probes specific for the *Iel* insertion element of *S. enteritidis* on their surface), and allowed to hybridize to create a sandwich structure due to their specificity (Figure 1). Following hybridization, sandwich structures were isolated and washed using magnetic separation, and then added to individual electrode SPCE chips for DPV readout. Current peaks were observed between 0.30 and 0.35 V, the reduction peak of gold ions. We found that similar to the detection of PCR amplified DNAt (Figure 2A), the AuNP-DNA biosensor was also able to detect non-PCR amplified PBC extracted DNAt (Figure 2B). Although there was some variability between duplicate samples, the results demonstrated a trend in average differential peak values of 5.0 × 10^−7^ A, 6.0 × 10^−6^ A, and 1.1 × 10^−5^ A, for the various *S. enteritidis* PBC genomic DNAt concentrations (3 ng/μL, 1 ng/μL, and 0.1 ng/μL, respectively). In addition, the specificity of our AuNP-DNA biosensor was also revealed as the detection peak of our negative control, PCR amplified *B. anthracis* non-specific DNA (NS-DNA: 3.0 × 10^−6^, 0.1 ng/μL) was found to be comparable to that of the H_2_O blank (2.5 × 10^−6^ A) (Figure 2A). Surprisingly, a hook effect was observed at the highest DNAt concentration tested, 3 ng/μL, in both the PCR amplified and non-PCR amplified DNAt detection, with detection peaks occurring at lower levels than those produced from the lower DNAt concentrations tested.

**Figure 3.**
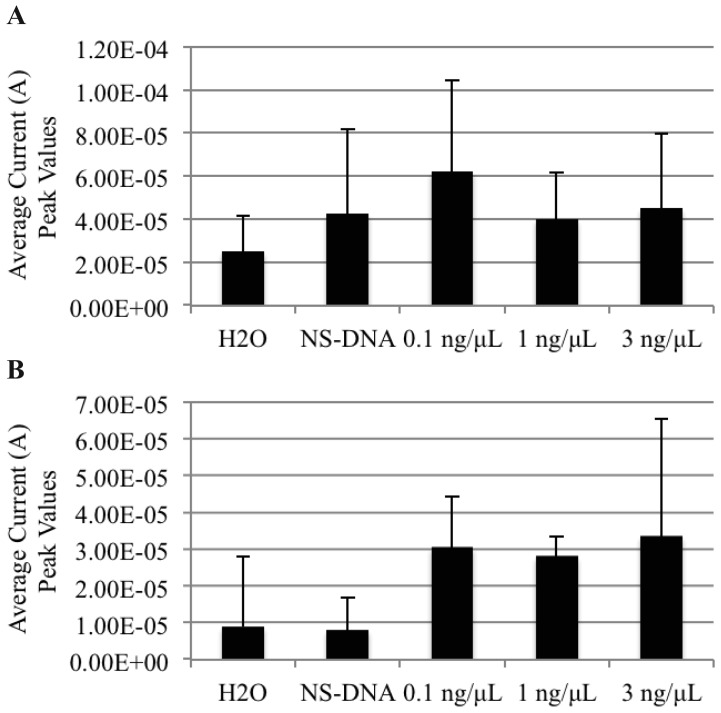
Non-PCR amplified *S. enteritidis* genomic DNAt can be detected from a mixed DNA culture using the AuNP-DNA biosensor. Graphs represent the average differential current peak values *vs.* DNAt concentration. (**A**) TRIzol^®^ extracted genomic DNAt from PBC and (**B**) phenol/ethanol extracted genomic DNAt from PBC. H_2_O, blank control; NS-DNA, non-specific PCR amplified *B. anthracis* DNAt (0.1 ng/μL) negative control; HCl, 1M hydrogen chloride. Graphs represent the average value of duplicate samples for each condition. Error bars represent the standard deviation of the mean.

As the AuNP-DNA biosensor demonstrated the ability to detect freshly extracted non-PCR amplified genomic DNAt from a single purified bacterial culture (PBC), we next sought to determine if the biosensor could detect non-PCR amplified genomic DNAt from a mixed bacterial culture (MBC) sample. Fresh, PBCs of *S. enteritidis, E. coli*, and *S. boydii* were grown individually overnight at 37 °C, and mixed the following day into a MBC. The genomic DNAt was then extracted from the MBC using both the TRIzol^®^ and phenol/ethanol extraction methods to generate the MBC DNAt, that was then hybridized with AuNPs and MNPs, and detected on the AuNP-DNA biosensor using DPV (Figure 3). Analogous to our previous findings using PBC, DNAt was distinguishable from the MBC extraction using both DNA extraction methods. Interestingly, we also observed a hook effect at the higher genomic DNAt concentration of 3 ng/μL, with a detection peak of 4.5 × 10^−5^ A, when using the TRIzol^®^ method.

### 3.3. AuNP-DNA Biosensor Detection of Genomic DNA Extracted from Liquid Food Matrices

**Figure 4.**
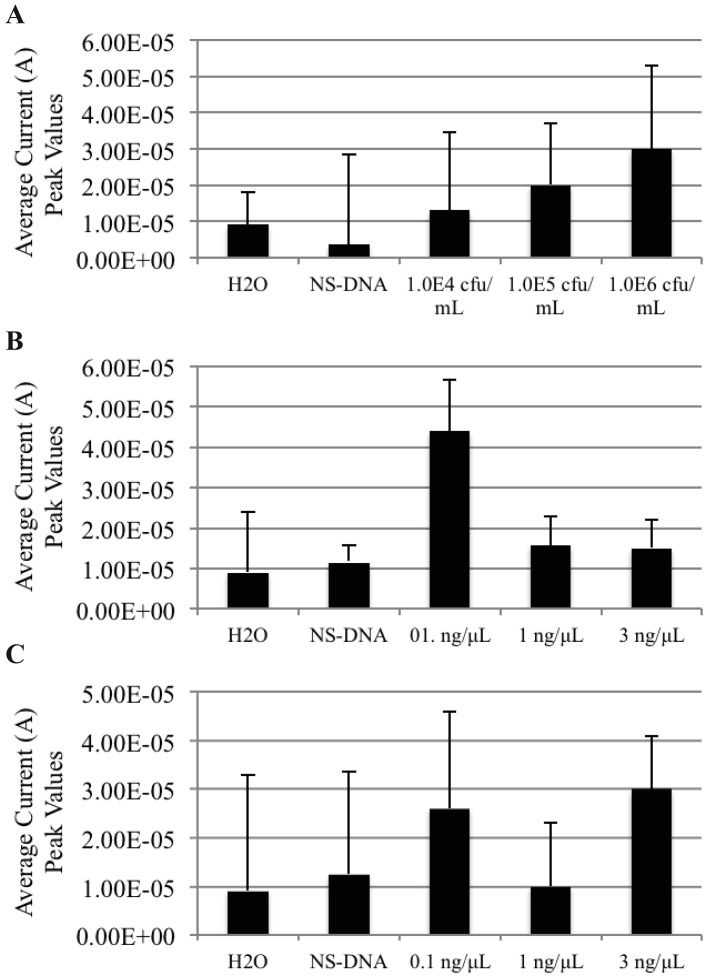
Non-PCR amplified *S. enteritidis* genomic DNAt can be detected in basic liquid food matrices using the AuNP-DNA biosensor. Graphs represent the average differential current peak values *vs.* DNAt concentration. (**A**) TRIzol^®^ extracted genomic DNAt from 2% milk spiked with PBC; (**B**) TRIzol^®^ extracted genomic DNAt from 2% milk spiked with MBC; (**C**) Phenol/ethanol extracted genomic DNAt from 2% milk spiked MBC. H_2_O, blank control; NS-DNA, non-specific PCR amplified *B. anthracis* DNAt (0.1 ng/μL) negative control; HCl, 1M hydrogen chloride. Graphs represent the average value of duplicate samples for each condition. Error bars represent the standard deviation of the mean.

**Figure 5.**
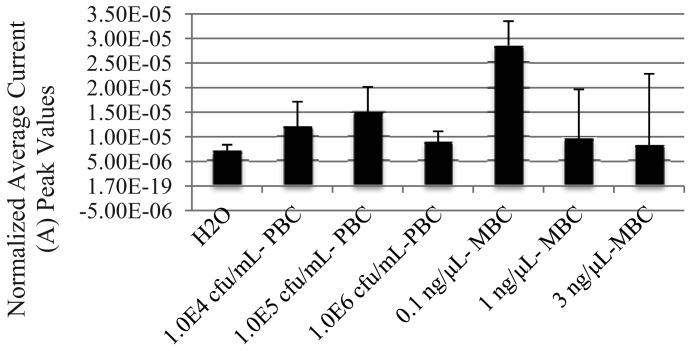
Non-PCR amplified *S. enteritidis* genomic DNAt can be detected in acidic liquid food matrices using the AuNP-DNA biosensor. Graphs represent the average differential current peak values *vs.* DNAt concentration attained from genomic DNAt extracted from PBC and MBC spiked orange juice. H_2_O, blank control; NS-DNA, non-specific PCR amplified *B. anthracis* DNAt (0.1 ng/μL) negative control; HCl, 1M hydrogen chloride. Graphs represent the average value of duplicate samples for each condition. Error bars represent the standard deviation of the mean.

As the AuNP-DNA biosensor exhibited a trend towards the detection of non-PCR amplified genomic DNAt from PBC and MBC samples, we next determined if it could also detect non-PCR amplified genomic DNAt extracted from basic and acidic liquid food matrices spiked/contaminated with *S. enteritidis* PBC or MBC. We first spiked 9 mL samples of 2% milk (a basic food matrix, Figure 4A) and 100% orange juice (an acidic food matrix, Figure 5) with 1.0 mL of fresh, PBC of *S. enteritidis* at three bacterial concentrations: 1.0^4^ cfu/mL (orange juice only), 1.0^5^ cfu/mL and 1.0^6^ cfu/mL. The spiked matrices were allowed to incubate at room temperature for 15 min before aliquoting a 1.0 mL sample of each for TRIzol^®^ extraction of genomic DNAt. The DNAt samples were then hybridized with AuNPs and MNPs, and detected on the AuNP-DNA biosensor using DPV. The results display that the AuNP-DNA biosensor was also able to detect *S. enteritidis* genomic DNAt from PBC spiked basic and acidic food matrices at all levels of contamination (Figures 4A and 5).

Following these results, we conducted similar contamination experiments using MBCs. Using 1.0^5^ cfu/mL of MBC to spike 9 mL aliquots of both basic and acidic liquid food matrices, we extracted DNAt using both TRIzol^®^ and the phenol/ethanol extraction methods for the basic liquid food matrix (Figures 4B and C), and only TRIzol^®^ for the acidic food matrix (Figure 5). As previously observed, the lowest concentration of DNAt used, 0.1 ng/μL, resulted in the best detection as shown by the highest peak produced under all conditions used (4.4 × 10^−5^ A TRIzol^®^ and 2.6 × 10^−5^ A phenol from milk, and 2.86 × 10^−5^ A TRIzol^®^ from orange juice). Likewise, the highest concentration tested, 3 ng/μL, resulted in the lowest peak detection values compared to both the 0.1 and 1 ng/μL concentrations from both of the TRIzol^®^ MBC extracted DNAt samples (basic 1.5 × 10^−5^ A and acidic 8.33 × 10^−6^ A).

## 4. Discussion

In our previous study using PCR amplification of DNAt, we reported a sensitivity range of 7–700 ng/mL of DNAt for single pathogen detection using a AuNP-DNA biosensor [16]. As the use of PCR equipment and reagents can be costly and cumbersome when used both in the laboratory and in field settings, it is important to transition the biosensor detection system into a more environmentally durable application that can be useful for a multitude of settings, such as field and resource limited laboratory conditions. With that focus, this is the first study to demonstrate the *proof-of-concept* that a AuNP-DNA biosensor can detect freshly extracted non-PCR amplified genomic DNAt from PBC and MBC conditions. Most importantly, the AuNP-DNA biosensor can also detect non-PCR amplified genomic DNAt from PBC and MBC contaminated basic and acidic liquid food matrices. Equally, our preliminary data suggest that the sensitivity of detection achieved in this study fell within the previously reported PCR amplified detection range [16], as all of our current experiments demonstrated the highest detection of the non-PCR amplified genomic DNAt to be at the concentration of 1 ng/μL or 100 ng/mL, albeit variability within triplicate samples.

Although, we were able to detect the non-PCR amplified genomic *S. enteritidis* DNAt from all three DNAt concentrations tested, we found that the detection peaks showed an inverse correlation with increasing concentration. That is, there appeared to be a loss of sensitivity as the non-PCR amplified extracted DNAt concentration increased, as shown by the lowest peaks corresponding to the highest concentration of 3 ng/μL (3,000 ng/mL) tested. These results suggest that a hook effect might be occurring as the concentration of DNAt product increases. This increase in DNAt product could result in the over saturation of both the DNAt capturing AuNPs and MNPs, and consequently, the inhibition of the sandwich structure formation. A hook effect is a common occurrence in many biochemical assays, such as quantitative real time PCR and ELISA assays [29–33], that require the formation of a sandwich-like structure between two capturing molecules and a target ligand. During a hook effect, the concentration of the target ligand (e.g., DNA, chemical, or protein antigen) greatly exceeds the concentration of the capturing molecule (e.g., complementary DNA or protein antibody). This overabundance of target ligand saturates all the binding sites of the capturing molecules and does not allow the formation of the sandwich structure, resulting in a decreased or no detection (false negative) outcome of the reporting system. While we believe this event is occurring at the higher concentrations tested, in naturally occurring applications, samples being tested with DNA-based biosensors would not contain such high concentrations of DNAt (1,000 ng/mL or higher), and will often contain concentrations that are much lower (below 500 ng/mL), as most PCR amplified DNA detection methods are validated within this lower range.

## 5. Conclusions

Our current study is a first step towards establishing the *proof-of-concept* that a AuNP-DNA biosensor can be used for the detection of genomic pathogenic DNAt in both PBC, MBC and contaminated liquid food matrices without the use of expensive, time consuming and cumbersome PCR amplification. It is imperative that the development of all biosensors includes a wide range of applicability, especially for resource limited and field laboratory conditions. Consequently, future efforts will focus on expanding upon these preliminary findings and investigating if detection levels of non-PCR amplified DNAt with these AuNP-DNA biosensors can be achieved using lower concentrations of DNAt (7–50 ng/mL) that are on par with the lower detection sensitivity of PCR amplified DNAt detection. Equally, future efforts will focus on establishing the statistical variability of the detection mechanism of the AuNP-DNA biosensor and refining the DNA extraction methods, in anticipation that the system can one day be used in field settings. Similarly, it will be necessary to repeat these experiments in other food matrices, including other basic and acidic liquid environments and solid food matrices prone to *S. enteritidis* contamination, in order to establish the wider applicability of the AuNP-DNA biosensor. Detection of would-be pathogens, such as *S. enteritidis, E. coli*, and *S. boydii*, do not only cause accidental foodborne illnesses, but can also be used as the causative agent of full blown epidemics if used as bioterrorism instruments. AuNP-DNA biosensors may serve as an excellent rapid detection system in the prevention of such events.
